# Prescribing Target Running Intensities for High-School Athletes: Can Forward and Backward Running Performance Be Autoregulated?

**DOI:** 10.3390/sports6030077

**Published:** 2018-08-09

**Authors:** Aaron Uthoff, Jon Oliver, John Cronin, Paul Winwood, Craig Harrison

**Affiliations:** 1 Sports Performance Research Institute New Zealand (SPRINZ), AUT Millennium, AUT University, 17 Anatres Pl, Rosedale, Auckland 0632, New Zealand; joliver@cardiffmet.ac.uk (J.O.); john.cronin@aut.ac.nz (J.C.); Paul.Winwood@toiohomai.ac.nz (P.W.); craig@athletedevelopment.org.nz (C.H.); 2 Youth Physical Development Centre, School of Sport, Cardiff Metropolitan University, Cyncoed Campus, Cyncoed Road, Cardiff CF23 6XD, UK; 3 School of Health and Medical Science, Edith Cowan University, Perth 6027, Australia; 4 Department of Sport and Recreation, School of Applied Science, Toi Ohomai Institute of Technology, Tauranga 3112, New Zealand

**Keywords:** peak height velocity, tempo training, retro-running, reliability

## Abstract

Target running intensities are prescribed to enhance sprint-running performance and progress injured athletes back into competition, yet is unknown whether running speed can be achieved using autoregulation. This study investigated the consistency of running intensities in adolescent athletes using autoregulation to self-select velocity. Thirty-four boys performed 20 m forward running (FR) and backward running (BR) trials at slow, moderate and fast intensities (40–55%, 60–75% and +90% maximum effort, respectively) on three occasions. Absolute and relative consistency was assessed using the coefficient of variation (CV) and intraclass correlation coefficients (ICC). Systematic changes in 10 and 20 m performance were identified between trials 1–2 for moderate and fast BR (*p* ≤ 0.01) and during moderate BR over 20 m across trials 2–3 (*p* ≤ 0.05). However, comparisons between trials 2–3 resulted in low typical percentage error (CV ≤ 4.3%) and very good to excellent relative consistency (ICC ≥ 0.87) for all running speeds and directions. Despite FR being significantly (*p* ≤ 0.01) faster than BR at slow (26%), moderate (28%) and fast intensities (26%), consistency was similar in both running directions and strongest at the fastest speeds. Following appropriate familiarization, youth athletes may use autoregulation to self-select prescribed FR and BR target running intensities.

## 1. Introduction

It is common for coaches to prescribe targeted running speeds (e.g., half maximum speed, three-quarter maximum speed, or maximum speed), or intensities (e.g., a percentage of maximal running effort), during warm-ups [1], in training [2,3] or for rehabilitation [4]. Target intensities may range from relatively slower, submaximal efforts [5], to fast, maximal efforts [6], depending on the desired outcome of a session. Based on where and how target speed training is utilized in an athlete development program, this training strategy may serve to prepare athletes for the rigors of competition, elicit desired training adaptations or help athletes return to their sport following injury. In the absence of sensory feedback, the capability of athletes to achieve target running intensities is facilitated by their ability to self-select their running velocity using auto-regulated strategies [7]. Although it is common for coaches to prescribe target running intensities, there is currently little evidence to support the idea that athletes are able to consistently achieve similar performances at these intensities between training sessions.

Submaximal target speeds (i.e., tempo running) have been programmed to improve running mechanics and promote aerobic adaptations [8,9], while target speeds at maximal or near maximal sprint-running efforts are used in training to reflect biomechanical and physiological demands similar to those experienced by many athletes participating in field and court sports [9,10]. Maximal effort forward sprint-running over short distances (i.e., 20 m) have been reported to have high inter-day reproducibility in paediatric populations with coefficient of variations (CV) around 2% and high intraclass correlational coefficients (ICC) between 0.82 and 0.91 [11,12], yet a paucity of information around the reproducibility of submaximal speeds exists in youth. Moreover, no such information exists around the consistency of backward running (BR) training in youth. 

Forward running (FR) and BR are sport-specific movements utilized by adults and adolescents during most over-ground sports [13,14]. However, the majority of scientific research has been on FR or forward sprint-running. This is interesting given that match analysis in youth football players has shown BR accounts for approximately 5% of total competition performance [15]. Recently, BR has been proposed as a method for enhancing athletic performance given its unique acute and longitudinal adaptations relative to FR [16]. Running speed during maximal efforts in adults have been reported to be approximately 30% slower during BR compared to FR [17], primarily as a result of shorter stride lengths and decreased reliance on the elastic components of the stretch-shortening cycle in BR [17,18,19]. At submaximal speeds, however, it is unknown whether similar decreases will be realized between the two running directions when asked to run at relative intensities. These biomechanical differences between FR and BR make them uniquely beneficial for inducing acute and long-term adaptations. Given BR’s distinct biomechanical profile and the dearth of scientific literature on this running direction, empirical research is necessary to guide prescription strategies for BR. 

Athletes use autoregulation to self-select targeted running speeds during both FR and BR in a variety of sports training situations, whether it be to prepare for the demands of competition or as a return to play protocol following injury [4,20]. However, the ability of high-school athletes to accurately self-select targeted running speeds, i.e., slow, moderate and fast, during either FR or BR, using auto-regulated strategies is unknown. Between session consistency has been reported for maximal effort forward sprint-running performance in youth athletes [11,12], yet no information about the consistency of running speeds exists at submaximal intensities in youth. Additionally, no information regarding the ability of athletes to run at prescribed target speeds is available on BR in young athletes. Therefore, the primary purpose of this study was to determine the ability of youth athletes to run at prescribed target speeds during short over-ground efforts. An additional aim of this research was to establish and compare velocities associated with FR and BR at different prescribed intensities. 

## 2. Materials and Methods

### 2.1. Participants

Thirty-four youth male athletes agreed to participate in this study. All participants were physically active and involved in summer sport(s) during the study duration, which generally consisted of two training sessions and one competitive game in a typical week. Maturity status was assessed using a non-invasive measuring technique recorded as age from peak height velocity (PHV), as predicted from anthropometric measures of body mass, standing height and sitting height [21]. The athletes were a mean age of 16.4 ± 0.9 years, with stature of 1.80 ± 0.05 m, body mass of 80.6 ± 12.6 kg and maturation of 2.8 ± 1.0 years from PHV. All parents/guardians provided written consent and assent from the participants was obtained prior to testing. The protocol was reviewed and approved by the Institutional Ethics Committee.

### 2.2. Measures

Running velocities associated with auto-regulated slow (i.e., 40–55% of maximal sprint performance), moderate (i.e., 60–75% of maximal sprint performance) and fast intensities (i.e., +90% of maximal sprint performance) served as the dependent variables. Double beam electronic photocell timing gates (Swift Performance Equipment, Wacol, Australia), linked to an electronic timer, were used to determine sprint times of the running trials under each condition. Running times were then used to calculate average velocities between 0–10 m and 0–20 m splits for all running trials. 

### 2.3. Design and Procedures

Testing was conducted on an outdoor turf field where weather conditions were consistently dry and runs were completed perpendicular to wind to ensure no tail or headwind. Wearing the same clothing and athletic training shoes, athletes were required to attend three consecutive testing sessions, seven days apart, at the same time of the day and under the same testing procedures. All participants attended a practice session prior to the first testing trial where they were familiarized with running at different target speeds via verbal feedback on their performance times following each 20 m run. Target speeds at slow, moderate and fast intensities were chosen to reflect running efforts at approximately half, three-quarters and near maximal speed [5]. During each testing session, athletes completed 3 × 20 m repetitions at each intensity in both running directions (i.e., 18 × 20 m total runs). Running trials were randomized in the first experimental session and athletes were tested in the exact same order on all other occasions. To minimise potential fatigue there was at least two minutes of passive rest between 20 m runs. A standardized warm-up was conducted before the familiarization and testing sessions. The warm-up consisted of progressively increased running intensities up to 90% of perceived maximal effort both forward and backward over 20 m, interspersed with dynamic stretching of the lower limbs. 

Athletes started in a split stance with their leading foot directly behind a tape placed 0.3 m behind the first gate and were prompted to run through the timing lights which were placed at the start, 10 m and 20 m marks. Timing gates were set at a height of 92.5 cm (top beam) and 68 cm (bottom beam) which corresponded closely with the approximate height of the athletes’ centre of mass. A 20 m trial was chosen to reflect common distances covered during warm-ups [22], as well as typical sprint and BR distances as reported from match performance analysis in youth footballers [15]. This research used a similar approach to Gabbett [11] who found that youth produced reliable maximal FR efforts over 10 m and 20 m (ICC = 0.82 and 0.91, respectively). The averaged running velocities from 0–10 m and 0–20 m for each running intensity was used to assess absolute and relative consistency of FR and BR. The corresponding running intensities for FR and BR trials were compared to assess the relationship between intensities for each running direction.

### 2.4. Statistical Analysis

Assumptions of normality and descriptive variables were quantified using IBM SPSS statistics (V.23.0). Data is presented as means with 95% confidence limits (CL). Pairwise analysis of reliability was investigated using averaged data over the three running trials between the first and second testing sessions and between the second and third testing sessions for each dependent variable. To determine whether velocities differed between days, a one-way analysis of variance using repeated measures was conducted for each relative running condition. A Bonferroni pairwise comparison was used to determine whether differences occurred between the testing sessions one to two and two to three. Performance data for each running condition was log transformed to properly estimate typical percentage errors (i.e., absolute consistency) expressed as mean percentage change and coefficient of variation (CV) [23]. Test-retest correlations were expressed as intraclass correlation coefficients (ICC’s, absolute agreement) using a two-way random model and average measures [24]. Typical error as a percentage was considered acceptable with CVs ≤ 10% [25]. ICC classification was considered as follows: ‘very poor’ (<0.20) ‘poor’ (0.20–0.49), ‘moderate (0.50–0.74), ‘good’ (0.75–0.90) or ‘excellent’ (>0.90) [26]. Running velocities were compared between relative FR and BR intensities over 10 m and 20 m using paired samples *t*-tests. To counteract the problem of multiple comparisons and the chance of a false positive, significance was accepted at the *p* ≤ 0.01 level.

## 3. Results

Pairwise comparisons between trials revealed systematic differences in mean 10 m and 20 m velocities for moderate BR (*p* ≤ 0.01) and fast BR (*p* ≤ 0.01) from trials 1–2 and moderate BR (*p* ≤ 0.01) between trials 2–3 over 20 m (see Table 1). Between trials 1–2 change in mean velocity ranged from −6.15 to 5.59% and between trials 2–3 the change in mean velocity was between ± 2.18% for all conditions. For all target speeds and running conditions, the change in the mean was smaller in trials 2–3 compared to trials 1–2.

In terms of absolute consistency, CVs ranged from 1 to 12%, with only slow FR over 10 and 20 m > 10%. As can be observed from Table 1, greater variability was associated between Trials 1–2 (average CV across all measures = 6.92%) as compared to trials 2–3 (average CV across all measures = 2.87%). It appears that variability decreased with increasing velocity for both FR and BR. In terms of absolute agreement, ICC ranged from 0.45 to 0.99. Lower absolute agreement was associated with trials 1–2 (average ICC across all measures = 0.67) as compared to trials 2–3 (average ICC across all measures = 0.93). In nearly all instances the magnitude of the improvement between consecutive pairs of trials for both the CV and ICC meant the confidence limits from comparing trials 1–2 and 2–3 did not overlap. Both CV and ICC were similar for FR and BR at all relative speeds and CV were lower at faster speeds for both running directions. 

**Figure 1:**
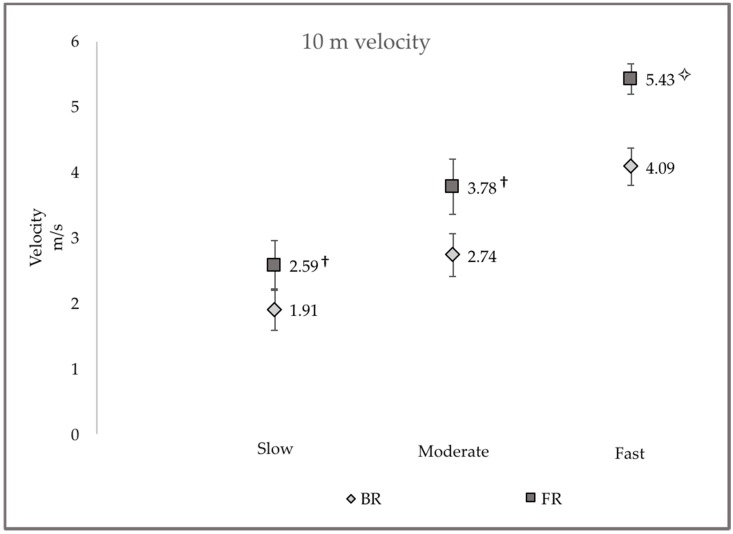
Comparison of averaged 10 m FR and BR velocities for athletes running at slow, moderate and fast intensities. ✧ = FR velocity significantly faster than BR velocity (*p* ≤ 0.01); † = FR velocity significantly faster than BR velocity (*p* ≤ 0.001).

**Figure 2:**
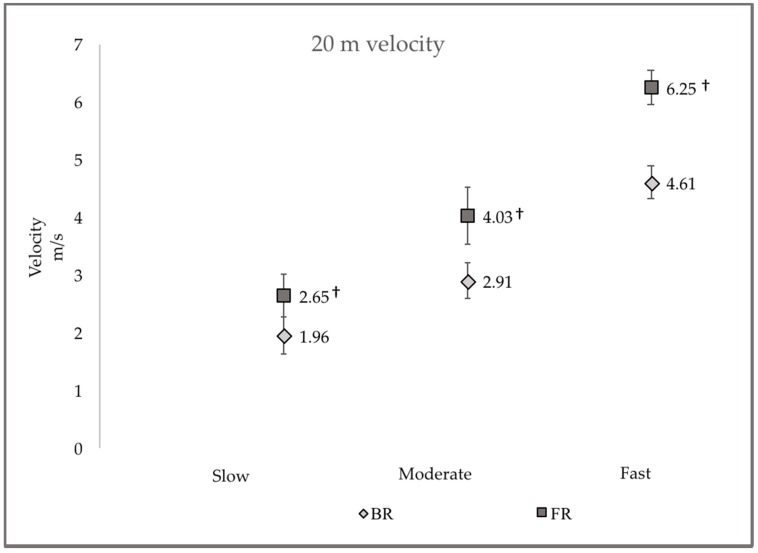
Comparison of averaged 20 m FR and BR velocities for athletes running at slow, moderate and fast intensities. † = FR velocity significantly faster than BR velocity (*p* ≤ 0.001).

Velocities for FR and BR showed similar trends of decreasing speed from the first to the second testing session at the slow pace (−4.86% and −3.98%) and increasing speed at moderate (4.72% and 5.43%) and fast intensities (0.77% and 2.26%), respectively. Average participant running velocity from all trials over 0–10 m and 0–20 m are presented in Figure 1 and Figure 2, respectively. Across all running intensities, significantly greater (*p* ≤ 0.01) FR speeds were observed to be 25–27% faster than BR over 10 m and 26–28% faster over 20 m. 

## 4. Discussion

The ability to autoregulate running speed is important in terms of preparing the body for sports (i.e., warm-up and training) in order to induce specific physiological and mechanical adaptations. The present study sought to quantify the ability of adolescent athletes to consistently achieve targeted speeds using autoregulation during FR and BR. The main findings of this study were: (1) Change in the mean, CVs and ICCs indicate that there was a systematic change between the first two trials, with better consistency in results in the latter trials, indicating a familiarization/learning effect; (2) athletes can autoregulate forward and backward running velocity consistently with adequate familiarization i.e., Trials 2–3—change in the mean < 2.2%; CV < 5%; ICC > 0.87; (3) greater absolute consistency and agreement was associated with greater velocity; (4) averaged FR velocity was approximately 27% greater than BR across all prescribed target intensities, however, consistency and agreement of performance within the relative speed zones (slow, moderate, fast) was similar. 

Improvements in the CV and ICC between trials 2–3 compared to trials 1–2 suggest that either a continued familiarization, or a learning effect, was present for both FR and BR across all intensities in trial 1, but learning or familiarizations ceased to continue in trials 2 and 3. The performance deviations reported in the present study are likely attributed to biological variations of the athletes since typical error associated with timing lights has been shown to be minimal [27]. The time around PHV has been associated with temporary disruption in coordination [28] and youth performances have been shown to fluctuate on some athletic tasks, such as a countermovement jump [29]. 

Despite the presence of systematic bias between testing occassions, the present research demonstrated that with ample familiarisation, youth athletes can consistently attain prescribed submaximal and maximal target speeds during FR and BR using autoregulation. This is important as coaches who prescribe target running intensities or use target speeds as a testing method must be confident that their exercises are training or measuring the appropriate athletic qualities [30,31]. Given the presence of a potential learning effect, youth athletes may require multiple familiarization sessions to become accustom to attaining consistent target running intensities via autoregulation. However, with an absence of literature on this topic, additional research is necessary to support this argument. 

Interestingly, as velocity increased, so did consistency of auto-regulated performances. Running at the fastest velocities were associated with the lowest error between days compared to slow and moderate efforts. Reliability statistics at the fastest intensities agree with previous findings that youth athletes can reproduce FR sprinting performances [11,12]. Up until now, maximal effort FR has been widely used as a performance assessment tool [32]. The findings herein suggest that maximal effort BR may be used similarly to maximal effort FR in order to evaluate performance as both running directions displayed low typical errors up to 20 m between sessions (CV = 0.99–1.42%) and high absolute agreement (ICC = 0.90–0.99) after ample familiarisation. While the present research provides promising information around the potential monitoring utility of both maximal effort FR and BR, direct comparisons to previous findings are difficult due to a scarcity of published literature related to the consistency of running direction and/or intensity using autoregulation in youth populations. 

Averaged FR velocity was approximately 27% greater than BR across all prescribed target intensities, however, consistency of selecting velocity within the relative speed zones (slow, moderate, fast) was similar. These velocity differences are in line with previous findings which demonstrated that submaximal and maximal running velocities during BR are, on average, 70% of FR velocity in adults [33]. Differences in velocity may be expected between the two running directions as the human body has evolved to run forwards and the lower limb joints are mechanically constrained by skeletal and soft tissue [34]. While BR appears to be less efficient at utilizing eccentric energy during the stretch-shorten cycle [35], this does not explain the similar consistencies achieved between the two running directions. As both running directions corrected velocity at the slowest intensity by becoming slower and moderate and fast velocities became faster between trials 1–2, how the inconsistencies in movement were resolved appears dependent on the speed of movement, rather than the running direction. According to motor programming theories and arguments of some researchers, each direction of movement is modulated by the same spinal neural network and modifications in one direction of locomotion may transfer to the other [19]. Theories of control models may help explain the current findings, yet current models are incomplete and contention exists around how each direction of running is accomplished [36]. 

Auto-regulating running velocity appears to be an important method for achieving target running intensities in athletic populations [7]. Understanding the consistency of performances between trials is essential for guiding exercise prescription and testing protocols. The present study identified that youth athletes can use autoregulation strategies to self-select a range of FR and BR speeds similarly in the absence of external cues. While the current study is limited to male athletes primarily post-PHV, it provides a focused understanding of gender- and maturity-specific performance for FR and BR at speeds used by practitioners and clinicians to prepare athletes for competition and progress back into their sport following injury. Knowing that greater differences in performance are experienced by less mature children when exposed to new stimuli compared to their more mature counterparts (e.g., adding resistance to FR) [37], scientists and practitioners would benefit from future investigations into the underlying mechanisms responsible for promoting motor control and the relationship between maturation and novel performance tasks in youth athletes. 

## 5. Conclusions

This is the first study to investigate the ability of youth athletes to use autoregulation as a means to self-select running velocity based on prescribed target running intensities. Running at target intensities is an exercise method which can be used to enhance athletic performance or progress injured athletes back into their sport. Therefore, practitioners must be confident that athletes are capable of selecting the desired running intensities in the absence of external sensory feedback. The present research demonstrated that youth athletes are able to employ auto-regulated strategies to consistently attain prescribed target running intensities both forward (CV ≤ 4.33%; ICC ≥ 0.87) and backward (CV ≤ 3.82%; ICC ≥ 0.92) following ample familiarization. The findings of this reasearch can be used to improve training or rehabilitation strategies, enhance adaptations and confidently track running performances at target intensities based on the needs of the athlete or demands of the sport. We suggest that the athlete’s familiarity with using autoregulation to self-select running velocities be taken into account when prescribing target running intensities for high-school aged athletes. 

## Figures and Tables

**Table 1:** Auto-regulated forward running (FR) and backward running (BR) velocities over 10 m and 20 m and associated consistency data for slow, moderate, and fast intensities.

Variable	Day 1 (m ± sd)	Day 2 (m ± sd)	Day 3 (m ± sd)	% Change in Mean		CV		ICC	
				Day 1–2 (95% CL)	Day 2–3 (95% CL)	Day 1–2 (95% CL)	Day 2–3 (95% CL)	Day 1–2 (95% CL)	Day 2–3 (95% CL)
10 m									
Slow forward (ms^−1^)	2.70 ± 0.40	2.53 ± 0.41	2.54 ± 0.31	−6.15 (−11.3 to 0.75)	0.65 (−1.43 to 2.78)	12.0 (9.58 to 16.09)	4.33 (3.48 to 5.47)	0.45 (0.14 to 0.68)	0.93 (0.82 to 0.95)
Slow backward (ms^−1^)	1.96 ± 0.33	1.87 ± 0.30	1.89 ± 0.29	−3.98 (−7.88 to 0.08)	1.07 (2.96 to 1.87)	8.75 (7.00 to 11.68)	3.82 (3.07 to 5.06)	0.75 (0.56 to 0.87)	0.94 (0.88 to 0.97)
Moderate forward (ms^−1^)	3.70 ± 0.49	3.84 ± 0.36	3.80 ± 0.39	4.40 (0.41 to 8.54)	−1.25 (−3.04 to 0.57)	8.21 (6.57 to 10.94)	3.78 (3.06 to 5.00)	0.56 (0.28 to 0.75)	0.87 (0.76 to 0.93)
Moderate backward (ms^−1^)	2.67 ± 0.32	2.81 ± 0.33	2.75 ± 0.30	5.27 ✧ (1.74 to 8.93)	−1.25 (−3.44 to −0.33)	7.16 (5.74 to 9.53)	3.26 (2.62 to 4.32)	0.67 (0.43 to 0.82)	0.92 (0.85 to 0.96)
Fast forward (ms^−1^)	5.38 ± 0.28	5.45 ± 0.22	5.45 ± 0.20	1.49 (−0.23 to 3.24)	−0.13 (−0.74 to 0.50)	3.52 (2.83 to 4.66)	1.24 (1.00 to 1.64)	0.48 (0.18 to 0.70)	0.90 (0.81 to 0.95)
Fast backward (ms^−1^)	4.02 ± 0.32	4.11 ± 0.28	4.13 ± 0.27	2.36 ✧ (0.95 to 3.78)	0.34 (−0.36 to 1.04)	2.85 (2.29 to 3.76)	1.42 (1.14 to 1.87)	0.87 (0.75 to 0.93)	0.96 (0.92 to 0.98)
20 m									
Slow forward (ms^−1^)	2.72 ± 0.41	2.63 ± 0.40	2.60 ± 0.31	−3.57 (−8.39 to 1.51)	−0.75 (−2.76 to 1.3)	10.95 (8.74 to 14.66)	4.24 (3.40 to 5.61)	0.52 (0.23 to 0.73)	0.91 (0.82 to 0.95)
Slow backward (ms^−1^)	1.99 ± 0.35	1.94 ± 0.22	1.94 ± 0.25	−2.06 (−6.12 to 2.16)	0.20 (−1.54 to 1.98)	8.92 (7.14 to 11.91)	3.63 (2.92 to 4.81)	0.70 (0.48 to 0.84)	0.92 (0.85 to 0.96)
Moderate forward (ms^−1^)	3.94 ± 0.57	4.12 ± 0.43	4.04 ± 0.47	5.05 (0.96 to 9.30)	−2.18 (−4.03 to −0.30)	8.38 (6.70 to 11.17)	3.63 (3.17 to 5.23)	0.63 (0.37 to 0.80)	0.89 (0.79 to 0.94)
Moderate backward (ms^−1^)	2.83 ± 0.38	2.98 ± 0.37	2.92 ± 0.36	5.59 ✧ (2.06 to 9.24)	−2.18 ✧ (−3.64 to 0.70)	7.14 (5.72 to 9.50)	3.09 (2.49 to 4.09)	0.72 (0.51 to 0.85)	0.94 (0.89 to 0.97)
Fast forward (ms^−1^)	6.23 ± 0.33	6.26 ± 0.28	6.26 ± 0.27	0.57 (−0.64 to 1.79)	−0.05 (−0.54 to 0.44)	2.48 (1.99 to 3.27)	1.00 (0.81 to 1.32)	0.76 (0.57 to 0.87)	0.95 (0.91 to 0.98)
Fast backward (ms^−1^)	4.55 ± 0.40	4.64 ± 0.36	4.65 ± 0.35	2.16 ✧ (0.85 to 3.49)	0.15 (−0.32 to 0.64)	2.66 (2.14 to 3.51)	0.99 (0.80 to 1.30)	0.91 (0.82 to 0.95)	0.99 (0.97 to 0.99)

NB. ✧ Significant (*p* ≤ 0.01) for between day performances.

## References

[1] LaBella C.R., Huxford M.R., Grissom J., Kim K.Y., Peng J., Christoffel K.K. (2011). Effect of neuromuscular warm-up on injuries in female soccer and basketball athletes in urban public high school. Arch. Pediatr. Adolesc. Med..

[2] Bompa T.O. (2000). Total Training for Young Champions.

[3] Haugen T., Tonnessen E., Leirstein S., Hem E., Seiler S. (2014). Not quite so fast: Effect of training at 90% sprint speed on maximal and repeated-sprint ability in soccer players. J. Sports Sci..

[4] Heiderscheit B.C., Sherry M.A., Silder A., Chumanov E.S., Thelen D.G. (2010). Hamstring strain injuries: Recommendations for diagnosis, rehabilitation, and injury prevention. J. Orthop. Sports Phys. Ther..

[5] Perrier E.T., Pavol M.J., Hoffman M.A. (2011). The acute effects of a warm-up including static or dynamic stretching on countermovement jump height, reaction time, and flexibility. J. Strength Cond. Res..

[6] Lockie R.G., Murphy A.J., Schultz A.B., Knight T.J., de Jonge X.A.J. (2012). The effects of different speed training protocols on sprint acceleration kinematics and muscle strength and power in field sport athletes. J. Strength Cond. Res..

[7] Abbiss C.R., Laursen P.B. (2008). Describing and understanding pacing strategies during athletic competition. Sports Med..

[8] Francis C. (1997). Training for Speed.

[9] Gambetta V. (2007). Athletic Development: The Art & Science of Funcitonal Sports Conditioning.

[10] Arazi H., Keihaniyan A., EatemadyBoroujeni A., Oftade A., Takhsha S., Asadi A., Ramirez-Campillo R. (2017). Effects of hear rate vs. speed-based high intensity interval training on aerobic and anaerobic capacity of female soccer players. Sports.

[11] Gabbett T.J. (2002). Physiological characteristics of junior and senior rugby league players. Br. J. Sports Med..

[12] Oliver J., Williams A.C., Armstrong N. (2006). Reliability of a Field and Laboratory Test of Repeated Sprint Ability. Pediatr. Exerc. Sci..

[13] Bates B.T., Morrison E., Hamill J. (1984). A comparison between forward and backward running. The 1984 Olympic Scientific Congress Proceedings: Biomechanics.

[14] Gravina L., Gil S.M., Ruiz F., Zubero J., Gil J., Irazusta J. (2008). Anthropometric and physiological differences between first team and reserve soccer players aged 10–14 years at the beginning and end of the season. J. Strength Cond. Res..

[15] Rebelo A., Brito J., Seabra A., Oliveira J., Krustrup P. (2014). Physical match performance of youth football players in relation to physical capacity. Eur. J. Sport Sci..

[16] Uthoff A., Oliver J., Cronin J., Harrison C., Winwood P. (2018). A new direction to athletic performance: Understanding the acute and longitudinal responses to backward running. Sports Med..

[17] Weyand P.G., Sandell R.F., Prime D.N., Bundle M.W. (2010). The biological limits to running speed are imposed from the ground up. J. Appl. Physiol..

[18] Wright S., Weyand P.G. (2001). The application of ground force explains the energetic cost of running backward and forward. J. Exp. Biol..

[19] Mehdizadeh S., Arshi A.R., Davids K. (2015). Quantifying coordination and coordination variability in backward versus forward running: Implications for control of motion. Gait Posture.

[20] Van den Tillar R., Lerberg E., Von Heimburg E. (2016). Comparison of three types of warm-up upon sprint ability in experienced soccer players. J. Sport Health Sci..

[21] Mirwald R.L., Baxter-Jones A.D., Bailey D.A., Beunen G.P. (2002). An assessment of maturity from anthropometric measures. Med. Sci. Sports Exerc..

[22] Soligard T., Myklebust G., Steffen K., Holme I., Silvers H., Bizzini M., Junge A., Dvorak J., Bahr R., Andersen T.E. (2008). Comprehensive warm-up programme to prevent injuries in young female footballers: Cluster randomised controlled trial. Br. Med. J..

[23] Hopkins W.G. A New View of Statistics. http://sportsci.org/resource/stats/.

[24] Koo T.K., Li M.Y. (2016). A guideline of selecting and reporting intraclass correlation coefficients for reliability research. J. Chiropr. Med..

[25] Lloyd R.S., Oliver J.L., Hughes M.G., Williams C.A. (2009). Reliability and validity of field-based measures of leg stiffness and reactive strength index in youths. J. Sports Sci..

[26] Buchheit M., Mendez-Villanueva A. (2013). Reliability and stability of anthropometric and performance measures in highly-trained young soccer players: Effect of age and maturation. J. Sports Sci..

[27] Cronin J.B., Templeton R.L. (2008). Timing light height affects sprint times. J. Strength Cond. Res..

[28] Quatman-Yates C.C., Quatman C.E., Meszaros A.J., Paterno M.V., Hewett T.E. (2012). A systematic review of sensorimotor function during adolescence: A developmental stage of increased motor awkwardness?. Br. J. Sports Med..

[29] Gerodimos V., Zafeiridis A., Perkos S., Dipla K., Manou V., Kellis S. (2008). The contribution of stretch-shortening cycle and arm-swing performance in children, adolescents, and adult basketball players. Pediatr. Exerc. Sci..

[30] Hopkins W.G. (2000). Measures of reliability in sports medicine and science. Sports Med..

[31] Little T., Williams A.G. (2006). Sutability of soccer training drills for endurance training. J. Strength Cond. Res..

[32] Meylan C., Cronin J., Oliver J., Hughes M. (2010). Talent identification in soccer: The role of maturity status on physical, physiological and technical characteristics. Int. J. Sports Sci. Coach..

[33] Arata A. (1999). Kinematic and Kinetic Evaluations of High Speed Backward Running.

[34] Mattson M.P. (2012). Evolutionary aspects of human exercise—Born to run purposefully. Ageing Res. Rev..

[35] Cavagna G.A., Legramandi M.A., La Torre A. (2011). Running backwards: Soft landing-hard takeoff, a less efficient rebound. Proc. Biol. Sci..

[36] Hoogkamer W., Meyns P., Duysens J. (2014). Steps forward in understanding backward gait: From basic circuits to rehabilitation. Exerc. Sports Sci. Rev..

[37] Rumpf M.C., Cronin J.B., Mohamad I.N., Mohamad S., Oliver J., Hughes M. (2014). Acute effects of sled towing on sprint time in male youth of different maturity status. Pediatr. Exerc. Sci..

